# 

**Published:** 1913-03

**Authors:** 


					﻿PUBLISHED MONTHLY.
SUBSCRIPTION $1.00
ATLANTA
JOURNAL-RECCOCC
OF MECICICt/'C
¡Atlanta Medical and Surgical Journal, Established 1855. ) dilL Vann
and i^uUwen Medicci Reeord, E^tatjlishhd 1870.	jj/lil IL0!
Vol. LIX.
Atlanta, Ga., March, 1913.
No. 12
				

